# Comprehensive screening for immunodeficiency-associated vaccine-derived poliovirus: an essential oral poliovirus vaccine cessation risk management strategy

**DOI:** 10.1017/S0950268816002302

**Published:** 2016-10-20

**Authors:** R. J. DUINTJER TEBBENS, K. M. THOMPSON

**Affiliations:** Kid Risk, Inc., Orlando, FL, USA

**Keywords:** Antiviral drugs, health economics, mathematical modelling, polio, oral poliovirus vaccine

## Abstract

If the world can successfully control all outbreaks of circulating vaccine-derived poliovirus that may occur soon after global oral poliovirus vaccine (OPV) cessation, then immunodeficiency-associated vaccine-derived polioviruses (iVDPVs) from rare and mostly asymptomatic long-term excretors (defined as ⩾6 months of excretion) will become the main source of potential poliovirus outbreaks for as long as iVDPV excretion continues. Using existing models of global iVDPV prevalence and global long-term poliovirus risk management, we explore the implications of uncertainties related to iVDPV risks, including the ability to identify asymptomatic iVDPV excretors to treat with polio antiviral drugs (PAVDs) and the transmissibility of iVDPVs. The expected benefits of expanded screening to identify and treat long-term iVDPV excretors with PAVDs range from US$0.7 to 1.5 billion with the identification of 25–90% of asymptomatic long-term iVDPV excretors, respectively. However, these estimates depend strongly on assumptions about the transmissibility of iVDPVs and model inputs affecting the global iVDPV prevalence. For example, the expected benefits may decrease to as low as US$260 million with the identification of 90% of asymptomatic iVDPV excretors if iVDPVs behave and transmit like partially reverted viruses instead of fully reverted viruses. Comprehensive screening for iVDPVs will reduce uncertainties and maximize the expected benefits of PAVD use.

## INTRODUCTION

Extensive use of the oral poliovirus vaccine (OPV) led to the certified global eradication of wild poliovirus (WPV) of one of three serotypes (i.e. serotype 2) [1], apparent interruption of all serotype 3 WPV (WPV3) transmission [2], and confinement of indigenous serotype 1 WPV (WPV1) transmission to Pakistan, Afghanistan, and Nigeria by 2016 [3]. However, OPV can cause vaccine-associated paralytic poliovirus (VAPP) and evolve to vaccine-derived polioviruses (VDPVs) that can cause outbreaks similar to WPV outbreaks [4]. To end all poliomyelitis disease (i.e. polio), the world stopped all serotype 2-containing OPV use in 2016 and will stop the remaining two serotypes after assurance of global WPV1 and WPV3 eradication [5], leaving the inactivated poliovirus vaccine (IPV) as the only vaccine available to induce individual protection from polio. IPV provides much less protection from participation in faecal–oral transmission than OPV [6–9]. Consequently, in populations with conditions conducive to faecal–oral poliovirus transmission and/or low routine immunization coverage, population immunity to transmission will decrease rapidly after OPV cessation [10, 11]. OPV intensification prior to OPV cessation, appropriate synchronization and coordination, and aggressive outbreak response can prevent or control circulating VDPV (cVDPV) outbreaks after OPV cessation [12–17], such that the world would subsequently enter an unprecedented era with respect to population immunity to poliovirus transmission. Any outbreaks will become increasingly difficult to contain and OPV use for outbreak response will become increasingly risky due to the possibility of exporting OPV-related viruses to areas outside the target population that can support circulation and evolution of these viruses [13, 14, 17]. IPV likely cannot control a rapidly propagating outbreak in a setting conducive to intense faecal–oral poliovirus transmission [17, 18], and therefore long-term risk management should focus on prevention rather than relying on outbreak response preparedness.

Long-term poliovirus re-introduction risks include breaches in poliovirus containment and introductions of immunodeficiency-associated VDPVs (iVDPV) from rare (i.e. on the order of 100 identified worldwide since OPV use started in the 1960s) long-term poliovirus excretors (defined as ⩾6 months of excretion) with B-cell-related primary immunodeficiency diseases (PIDs) [19, 20]. Global poliovirus containment efforts and development of non-replicating IPV seed strains aim to minimize the risk of containment breaches [21–23]. Recognizing the iVDPV risks [24], ongoing efforts to develop polio antiviral drugs (PAVDs) led to Phase I and II clinical trials of one compound (pocapavir) and the development of other compounds that may work as a combined therapy to minimize drug resistance [25]. An effective PAVD would offer identified long-term iVDPV excretors protection from developing VAPP by clearing their infection, and this would reduce the risk of iVDPV introductions. However, the identification of iVDPV excretors remains a major challenge because the majority experience no polio symptoms during most or all of their infection [4, 26–29]. Although multiple largely cross-sectional screening studies conducted to date examined ~1000 PID patients for asymptomatic iVDPV excretion, this represents only a fraction of the estimated 10 000s of PID patients likely alive today [30]. Consequently, most long-term iVDPV excretors identified to date presented with VAPP through the global acute flaccid paralysis surveillance system [4]. Identifying a large fraction of asymptomatic long-term iVDPV excretors will require substantial additional efforts to comprehensively screen PID patients for iVDPVs. Unlike B-cell related primary immunodeficiencies, T-cell immunodeficiency diseases (e.g. HIV) do not appear to lead to long-term iVDPV infections [31, 32].

A global iVDPV prevalence model identified multiple uncertainties that limit our understanding of iVDPV risks [26]. To help inform decisions related to investments in PAVD development and use, we use existing models [23, 26] to explore the expected health and economic benefits of efforts to identify asymptomatic long-term iVDPV excretors and the role of key uncertainties related to iVDPV risks.

## METHODS

The discrete-event simulation model (i.e. the DES model) estimates the global prevalence of long-term iVDPV excretors as a function of time after OPV cessation [26]. It probabilistically simulates relevant monthly events over the lifetime of all global PID patients, including (i) birth, (ii) PID onset, diagnosis, and treatment, (iii) OPV infections, recovery, and VAPP, and (iv) death [26]. To characterize the variability between countries, the DES model adopts the stratification of the world into 710 populations with different basic reproductive numbers (*R*_0_ values) from an integrated global model of long-term risk management (i.e. the global model) [23]. The DES model uses these *R*_0_ values as a proxy for hygienic conditions that affect PID survival, with higher *R*_0_ values implying shorter PID survival. Each stochastic DES model iteration produces a different realization of long-term iVDPV excretors over time in each global model population, which may result in iVDPV introductions into the corresponding populations. The global model integrates multiple components: (i) polio vaccination policy choices, including the global switch from trivalent to bivalent OPV that occurred in late April and early May 2016 and the global cessation of the remaining two OPV serotypes assuming this will occur in 2019, (ii) poliovirus transmission and OPV evolution dynamics [33], (iii) economic inputs related to vaccination costs and the direct and indirect costs associated with polio cases, (iv) stochastic risks after OPV cessation (including iVDPV introductions based on iVDPV prevalence from the DES model), (v) characterization of the global variability in conditions (e.g. *R*_0_, vaccination coverage) and policies, and (vi) random poliovirus exportations between the 710 populations (structured into epidemiological blocks of 10 subpopulations each and nine larger geographical regions consisting of multiple blocks) [17, 23]. The global model evolves OPV-related viruses from the OPV parent strain (stage 0) given to and excreted by vaccine recipients through a 20-stage reversion process to fully reverted VDPVs (stage 19) for which we assume an equal *R*_0_ and paralysis-to-infection ratio (PIR) as typical homotypic WPVs [33]. The global model also tracks all PID patients from the DES model to account for the possibility that monovalent OPV (mOPV) use for outbreak response could generate new long-term iVDPV infections after OPV cessation. Introductions may or may not lead to sufficient initial transmissions to generate an outbreak, which the global model accounts for by randomly determining whether an introduction starts population-wide transmission (i.e. defined as an effective introduction) based on population immunity to transmission and the *R*_0_ of the introduced virus. Thus, populations at highest risk of outbreaks given an iVDPV introduction coincide with those in which PID survival remains the shortest.

An economic analysis used the global model to estimate the expected incremental net benefits of major long-term risk management policies involving OPV cessation compared to continued OPV use with or without continued supplemental immunization activities (SIAs) [23]. The base case assumed minimum global IPV use of at least one routine immunization dose for 5 years after cessation of the last OPV serotype in 2019 (policy abbreviation IPV5). The policy specifically assumes that populations that already used IPV in 2013 and upper middle-income populations (which we assumed switch to a three-dose IPV schedule at cessation of the last OPV serotype) continue to use three-dose IPV routine immunization schedules until the end of the analytical time horizon that extends for 40 years after the most recent Global Polio Eradication Initiate Strategic Plan took effect (i.e. *T*_end_ = 2053 [5]). In addition to IPV use and sufficient OPV intensification before OPV cessation of each serotype to prevent cVDPV outbreaks, the global model base case assumed aggressive outbreak response and relatively low risks of non-iVDPV long-term poliovirus re-introductions. Recognizing the risks that even the attenuated strains in OPV could establish circulation when population immunity to poliovirus transmission becomes very low and that widespread OPV use could start new long-term iVDPV infections, the outbreak response strategy assumed mOPV use for only the first 5 years after homotypic OPV cessation and IPV thereafter. In the rare event of uncontrolled outbreaks after OPV cessation, rather than continuing reactive outbreak response SIAs with IPV, the global model assumed that reaching an arbitrary threshold of 50 000 polio cases globally would lead to resumed OPV use in all countries that used OPV as of 2013 and that from then on the world would incur the expected costs and cases associated with OPV use in routine immunization and any SIAs. We refer to stochastic iterations in which this occurs as OPV restart iterations [23]. Variations around the base case without PAVD use included two scenarios of PAVD use starting on 1 January 2017. The first variation (PAVD40% scenario) assumed 40% PAVD effectiveness and that 50% of long-term iVDPV excretors who presented clinically with VAPP receive PAVDs. The second variation (PAVD90% scenario) assumed high PAVD effectiveness of 90% and that 90% of all long-term iVDPV excretors (including those with no paralytic symptoms) receive PAVDs.

This study first performs a probabilistic uncertainty and sensitivity analysis of the DES model (see Supplementary material section S1). We then consider in more detail the role of screening for asymptomatic long-term iVDPV excretors, assuming the global model base case for all other assumptions and a stratified set of 120 iterations (see Supplementary material section S2). Assuming a 90% effective combined PAVD therapy and that the existing acute flaccid paralysis surveillance system would allow PAVD administration to 90% of long-term iVDPV excretors at VAPP onset, we vary the fraction of asymptomatic long-term iVDPV excretors identified through the screening programme and receiving PAVDs [i.e. the identification fraction (IF)] between 25% and 90%. For each IF, we report global outcomes related to iVDPV risks and the health-economic benefits of the PAVD use. We do not include the costs of the iVDPV screening efforts. Consistent with the global model, we expressed monetary amounts in 2013 US dollars ($) and used a 3% discount rate.

We also consider the uncertainty about the transmissibility and neurovirulence of iVDPVs. While our prior work consistently assumed the same *R*_0_ and PIR for cVDPVs and iVDPVs as homotypic WPVs because no direct evidence exists to support lower values for VDPVs [20, 26, 33, 34], some possibility exists that iVDPVs could behave differently due to antigenic adaptations that may occur during prolonged replication in limited intestinal sites of a single host. Although the absence of any known outbreak despite over 70 known long-term iVDPV excretors to date reflects high surrounding population immunity to transmission through vaccination, it could also partly reflect lower *R*_0_ values for iVDPVs than WPVs. To explore this possibility, we run the IPV5 policy both without PAVDs and with an IF of 90%, assuming that iVDPV introductions into the subpopulation that the excretor resides in occur at partially reverted stage 10 instead of the fully reverted VDPV stage (stage 19). This implies that any iVDPV introductions start with an *R*_0_ and log PIR about halfway between those of the OPV parent strain and homotypic WPVs [33]. The lower *R*_0_ decreases the chances that the introduction leads to an outbreak, but it does not preclude further evolution of these viruses as they circulate until they reach the fully reverted stage.

## RESULTS

Table 1 shows the relative importance of uncertain inputs based on 1000 DES model iterations, with rank correlations closer to 1 (−1) indicating a stronger increasing (decreasing) effect of an input on the time until the last long-term iVDPV excretor stops excreting (Supplementary material section S1). Globally, the last excretor stops excreting in early 2028 on average (s.d. = 6·0 years) in the DES model with all uncertain inputs at their base case values. With the input uncertainty included, this average time increases by 4·4 years to mid-2032 (s.d. = 9·8 years). Uncertainty about the average duration of long-term iVDPV infections contributes most to the uncertainty about the time until the last iVDPV excretor in the world stops excreting. However, this results mainly from the possibility that a chronic excretor in a high-income country survives and remains infected for decades after OPV cessation, which represents a lesser concern globally because of the expected continued high IPV-induced population immunity to transmission in those countries. In low- and middle-income countries, in which population immunity to transmission will decrease to unprecedented low levels after OPV cessation [23], the uncertainty about the time until the last iVDPV excretor stops excreting depends most on the uncertainty about PID survival, followed by the potential long-term excretion probability and incidence of PID-predisposed births. Other inputs in Table 1 matter less because they primarily affect how many long-term iVDPV excretors exist at the time of OPV cessation with no or little effect on how long they subsequently remain infected.

**Table 1.** The contribution of key inputs to the uncertainty in the DES model [26], ranked by absolute values of the rank correlation between each input and the time until the last iVDPV excretor anywhere in the world stops excreting in the DES model

DES model input	Rank correlation between given model input and time when last iVDPV excretor stops excreting in				
	World	Low-income countries	Lower middle-income countries	Upper middle-income countries	High-income countries
Average duration of iVDPV infection (years)	0·49	0·10	0·12	0·11	0·56
Relative monthly death rate *vs*. baseline	−0·48	−0·66	−0·65	−0·68	−0·35
Potential long-term excretion probability	0·39	0·45	0·46	0·43	0·41
PID predisposition probability per birth	0·18	0·16	0·15	0·17	0·18
Increase in all OPV exposure rates	0·07	0·07	0·10	0·07	0·07
Relative probability of long-term OPV infection if treated *vs*. not treated	−0·06	−0·04	−0·05	−0·07	−0·04
Monthly PID onset probability	0·00	0·07	0·02	−0·01	0·02

DES, Discrete-event simulation; iVDPV, immunodeficiency-associated vaccine-derived poliovirus; OPV, oral poliovirus vaccine; PID, primary immunodeficiency disease.

Table 2 shows the effect of efforts to identify and treat asymptomatic long-term iVDPV excretors with PAVDs on various global model outcomes and reveals a consistent reduction in undesirable outcomes with increasing IF. Specifically, a higher IF means that more long-term iVDPV excretors clear their infection after receiving PAVDs, which reduces the expected number of iVDPV introductions, including from any long-term iVDPV excretors affected by mOPV used for outbreak response. This reduces the probability of outbreaks, including uncontrolled outbreaks leading to OPV restarts (see Supplementary material section S2 for characteristics of OPV restart iterations). Increased PAVD use also reduces the number of doses required for outbreak response SIAs, the probability of unmet stockpile vaccine needs and associated additional (uncontrolled) outbreaks [17]. Fewer outbreak response SIAs lowers the costs for outbreak response and results in expected financial savings between $250–520 million (undiscounted) for IFs from 0·25 to 0·90, mainly by avoiding perhaps unrealistically large numbers of IPV outbreak response SIAs that precede OPV restarts. The reduction in outbreak response SIAs with mOPV also reduces the number of PID patients infected by mOPV that develop new long-term iVDPV infections.

**Table 2.** Global model results for different assumptions about the IF, based on a stratified set of 120 stochastic iterations

Global model outcome	Base case (i.e. no PAVDs)	IF = 25% (decrease *vs*. base case)	IF = 50% (decrease *vs*. base case)	IF = 75% (decrease *vs*. base case)	IF = 90% (decrease *vs*. base case)
Average* number of effective iVDPV introductions					
From iVDPV excretors infected by mOPV SIAs	0·18	0·018 (0·16)	0·0030 (0·17)	0 (0·18)	0 (0·18)
From all iVDPV excretors	16	12 (4·7)	9·2 (7·3)	6·1 (10)	5·0 (12)
Probability of at least one outbreak	0·95	0·91 (0·049)	0·81 (0·14)	0·66 (0·30)	0·56 (0·40)
OPV restart probability	0·057	0·036 (0·021)	0·032 (0·025)	0·019 (0·038)	0·013 (0·044)
Average number of outbreak response SIA doses used (millions)					
mOPV	150	110 (38)	92 (58)	47 (100)	27 (120)
IPV	780	440 (340)	410 (370)	180 (600)	98 (680)
Both	930	550 (380)	500 (430)	230 (700)	120 (810)
Outbreak response SIA costs ($ millions)					
mOPV	100	75 (26)	61 (41)	32 (70)	18 (83)
IPV	510	290 (220)	270 (240)	123 (380)	70 (440)
Both	610	360 (250)	330 (280)	150 (450)	88 (520)
Average number of new long-term iVDPV infections	1·2	0·91 (0·31)	0·69 (0·52)	0·45 (0·77)	0·26 (0·95)
Average number of polio cases from 2016 on (thousands)					
57 iterations with OPV restart without SIAs†	1100	660 (400)	600 (460)	340 (730)	230 (840)
57 iterations with OPV restart with SIAs†	200	130 (71)	120 (77)	68 (130)	47 (150)
63 iterations without OPV restart	0·57	0·50 (0·071)	0·48 (0·089)	0·41 (0·16)	0·38 (0·19)
All iterations, assuming OPV restarts without SIAs	61	38 (23)	35 (26)	20 (41)	13 (48)
All iterations, assuming OPV restarts with SIAs	12	7·8 (4·1)	7·5 (4·5)	4·3 (7·6)	3·1 (8·9)
Incremental net benefits ($ billions) of IPV5 compared to:					
RC no SIAs, assuming OPV restarts without SIAs	12	13 (−0·72)	13 (−0·81)	14 (−1·3)	14 (−1·5)
RC with SIAs, assuming OPV restarts with SIAs	16	16 (−0·79)	16 (−0·79)	17 (−1·3)	17 (−1·5)

IF, Identification fraction; IPV, inactivated poliovirus vaccine; IPV5, baseline global policy of at least 5 years of IPV years after global OPV cessation of the last serotype; iVDPV, immunodeficiency-associated vaccine-derived poliovirus; mOPV, monovalent OPV; OPV, oral poliovirus vaccine; PAVD, polio antiviral drug; RC, reference case; SIA, supplemental immunization activity; $, year 2013 United States dollars.

* All averages represent weighted averages for the stratified set of iterations (see Supplementary material section S2).

† Includes all OPV restart iterations from the stratified set for all columns, such that with PAVDs the averages includes iterations both with and without restarts. The averages depend on whether we assume that the restart would involve resumption of OPV SIAs in addition to routine immunization.

Overall, by preventing outbreaks, PAVD use may avoid many OPV restarts that occur for the base case and the associated large numbers of polio cases [23]. While these iterations represent only 5·7% of all expected realizations (Supplementary material section S2), we observed reductions in average polio cases after 2016 even for the much more common iterations without OPV restarts. The percent reductions in these global model results remain somewhat limited because ~350 VAPP cases occur between 2016 and cessation of the last OPV serotype in 2019 regardless of PAVD use [23], but the absolute reductions in expected polio cases in iterations without OPV restart remain significant (i.e. 71 and 190 for IF = 25% and 90%, respectively). The decrease in polio cases and costs with PAVD use increases the incremental net benefits of OPV cessation compared to continued OPV use. For example, the expected incremental net benefits of IPV5 compared to the reference case of continued OPV use without SIAs increase from $12 billion without PAVDs to $13 billion with IF = 25–50% and to $14 billion with IF = 75–90%.

**Fig. 1.**
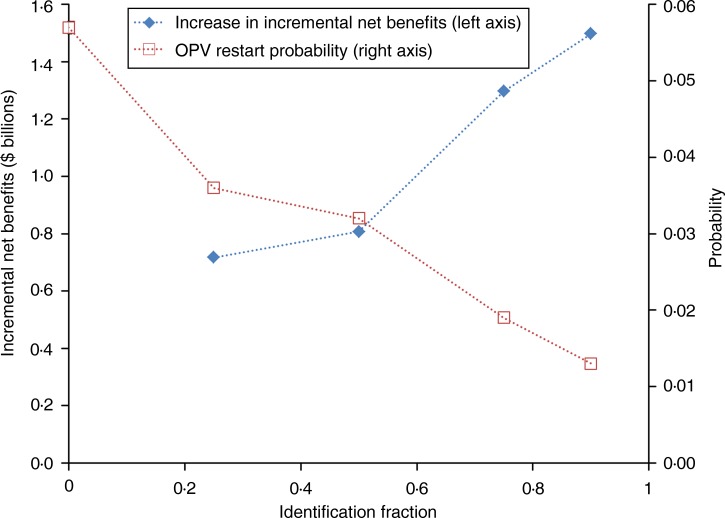
Relationship between identification fraction and oral poliovirus vaccine (OPV) restart probability (based on 57 OPV restart iterations) and the resulting increase in the incremental net benefits in year 2013 United States dollars ($) of the baseline policy of at least 5 years of inactivated poliovirus vaccine use after global cessation of the last OPV serotype compared to the reference case without supplemental immunization activities (base case OPV restart probability shown in figure as identification fraction of 0).

Figure 1 highlights the effect of the IF on the OPV restart probability and resulting increase in the incremental net benefits of IPV5 compared to the reference case without SIAs. These results do not include PAVD development or iVDPV screening costs, but they provide bounding estimates of the potential long-term benefits of making such investments. We estimate potential benefits of PAVDs ranging from $700 million (IF = 25%) to $1.5 billion (IF = 90%). However, these estimates depend on numerous assumptions in the model about outbreak consequences (e.g. outbreak response strategy and the frequency of poliovirus exportations to other blocks) and the relative importance of iVDPV risks compared to other risks (e.g. frequency of containment releases, pre-cessation OPV intensification).

Table 3 explores the effect of the uncertainty about the transmissibility of iVDPVs when introduced into the subpopulation in which the excretor resides. All prior results assumed iVDPVs behave like homotypic WPVs and cVDPVs when introduced into a subpopulation. If we assume instead that iVDPV strains did not acquire the same inherent transmissibility as homotypic WPVs but represent only partially-reverted VDPV virus (i.e. introducing them in reversion stage 10 instead of 19), then this markedly reduces iVDPV risks. First, the probability decreases that a contact between an iVDPV excretor and another individual in its subpopulation leads to an effective introduction that can start to transmit at the population level. Second, even effective introductions may die out before substantial circulation due to seasonality or other outbreak kinetics [14], or may stop more easily after an aggressive outbreak response. Consequently, introducing iVDPVs as partially reverted viruses yields large reductions in the probability of outbreaks and OPV restarts, expected outbreak response vaccine needs, and expected polio cases, which translates into higher incremental net benefits for both the base case without PAVDs and for the option with PAVDs. The benefits of PAVD use with IF = 90% decrease from $1.5 billion with fully reverted iVDPV introductions to $260 million with partially reverted iVDPV introductions.

**Table 3.** Global model results for different assumptions about the reversion stage of iVDPVs at the time of introduction into the subpopulation that the excretor resides in, based on a stratified set of 120 stochastic iterations

Global model outcome	Base case (no PAVDs), with iVDPV introductions in given reversion stage		IF = 90%, iVDPV introduction in given reversion stage (decrease *vs*. base case with same reversion stage assumption)	
	19 (fully reverted)	10 (partially reverted)	19 (fully reverted)	10 (partially reverted)
Average* number of effective iVDPV introductions				
From iVDPV excretors infected by mOPV	0·18	0·031	0 (0·18)	0 (0·031)
From all iVDPV excretors	16	17†	5·0 (12)	5·0‡ (12)
Probability of at least one outbreak	0·95	0·17	0·56 (0·40)	0·071 (0·095)
OPV restart probability	0·057	0·014	0·013 (0·044)	0·007 (0·007)
Average number of outbreak response SIA doses used (millions)				
mOPV	150	27	27 (120)	0·86 (26)
IPV	780	170	100 (680)	52 (120)
Both	930	200	120 (810)	53 (140)
Outbreak response SIA costs ($ millions)				
mOPV	100	17	18 (83)	0·53 (16)
IPV	510	113	70 (440)	41 (72)
Both	610	130	88 (520)	41 (89)
Average number of new long-term iVDPV infections	1·2	0·20	0·26 (0·95)	0 (0·20)
Average number of polio cases from 2016 on (thousands)				
57 iterations with OPV restart without SIAs‡	1100	240	230 (840)	110 (130)
57 iterations with OPV restart with SIAs‡	200	49	47 (150)	30 (19)
63 iterations without OPV restart	0·57	0·47	0·38 (0·19)	0·37 (0·092)
All iterations, assuming OPV restarts without SIAs	61	14	13 (48)	6·5 (7·7)
All iterations, assuming OPV restarts with SIAs	12	3·2	3·1 (8·9)	2·1 (1·2)
Incremental net benefits of IPV5 compared to ($ billions)				
RC no SIAs, assuming OPV restarts without SIAs	12	14	14 (−1·5)	14 (−0·26)
RC with SIAs, assuming OPV restarts with SIAs	16	17	17 (−1·5)	17 (−0·26)

IF, Identification fraction; IPV, inactivated poliovirus vaccine; IPV5, baseline global policy of at least 5 years of IPV years after global OPV cessation of the last serotype; iVDPV, immunodeficiency-associated vaccine-derived poliovirus; mOPV, monovalent OPV; OPV, oral poliovirus vaccine; PAVD, polio antiviral drug; RC, reference case; SIA, supplemental immunization activity; $, year 2013 United States dollars.

* All averages represent weighted averages for the stratified set of iterations (see Supplementary material section S2).

† The number of effective iVDPV introductions does not decrease for partially-reverted iVDPV introductions because the lack of substantial outbreaks associated with earlier iVDPVs introductions from the same or other long-term iVDPV excretors in the same population allows population immunity to continue to drop, which increases the probability that subsequent introductions become effective.

‡ Includes all OPV restart iterations from the stratified set for all columns, such that with PAVDs and/or partially reverted iVDPV introductions the averages includes iterations both with and without restarts. The averages depend on whether we assume that the restart would involve resumption of OPV SIAs in addition to routine immunization.

## DISCUSSION

This study explored several important uncertainties related to iVDPV risks after OPV cessation and the potential use of PAVDs to mitigate these risks. We find very high potential benefits of identifying asymptomatic long-term iVDPV excretors and treating them with effective PAVDs that may become available soon in the form of a combination therapy with at least two synergistic compounds [25]. These benefits range from an expected $700 million for an IF of 25% to $1.5 billion for an IF of 90% if iVDPVs possess the same transmission potential as WPVs and cVPDVs. The upper end represents a higher estimate than our previously reported estimate of $500 million based on an initial set of 100 global model iterations that included only one OPV restart associated with iVDPV outbreaks (i.e. probability of 1%) [23]. The estimate in this study draws from a much larger set of 1000 global model iterations, including 52 of 57 OPV restarts associated with iVDPV excretors (i.e. probability of 5.2%, see Supplementary material section S2).

The true benefits of PAVDs will depend on the actual realization of the uncertain future, with the observation of iVDPV2 outbreaks (or lack thereof) following the trivalent to bivalent OPV switch within the next few years already potentially providing some answers for serotype 2 that may inform decisions related to serotypes 1 and 3. Better understanding of the true transmissibility of iVDPVs would reduce uncertainty about the expected benefits of PAVD use. Resolving uncertainty about iVDPV prevalence (e.g. through better estimates of PID survival in developing countries) will affect the time until the last iVDPV excretor stops excreting in each income level, which will influence the potential number of cases prevented due to iVDPV outbreaks through the use of PAVDs. Other limitations and uncertainties from the global model [23] and the underlying poliovirus transmission and OPV evolution model [33] carry over to this analysis. Limitations and uncertainties that may particularly affect PAVD benefits include the kinetics of outbreaks (both during the initial stages following a point introduction [14] and the frequency of long-range poliovirus exportations [23]), the impact of IPV-alone on population immunity to transmission in developing countries [10, 35], the relationship between population immunity to transmission and the probability that an iVDPV or other introduction establishes transmission, and efforts to manage cVDPV [10, 12–14] and laboratory containment risks [20, 36, 37]. The actual duration of mOPV use for outbreak response and the availability of any new poliovirus vaccines with lower risks than OPV may further affect the risk of uncontrolled outbreaks due to iVDPVs and the benefits of PAVDs [38].

While the inherent transmissibility of iVDPVs remains very challenging to study [26], the assumption of equal transmissibility as homotypic WPV should remain the default assumption for the purposes of risk management unless evidence proves otherwise. All existing *in vitro* and animal data suggest no difference in phenotypic properties [4, 39], and widespread live poliovirus exposure in developing countries and/or IPV use in developed countries to date limit the epidemiological observability of iVDPV transmissibility. Further data collection of PID patients in developing countries and further screening for iVDPVs could reduce the uncertainty about: (i) PID survival rates, (ii) the probability that a PID develops a long-term infection, and (iii) the average duration of long-term iVDPV infections. The detection of a relatively high number of long-term iVDPV excretors from countries with high rates of consanguinity [4, 30, 40] may indicate that these countries experience a higher incidence of genetic predisposition to PIDs per birth, a higher risk of long-term iVDPV infections given OPV exposure for PIDs associated with consanguinity than other PIDs, higher OPV exposure rates, better PID survival, or some combination of these factors. Teasing out the possible explanations will require additional studies. Expanding iVDPV screening and continued development of PAVD combination therapies will take time and require resources. Further modeling may help characterize the costs and explore the impacts of different times to implement extensive PAVD use.

Despite the limitations and uncertainties, this study supports substantial investments in PAVDs and expanded screening and treatment of asymptomatic long-term iVDPV excretors as a key long-term polio risk management strategy. A growing network of physicians treating patients with PIDs [30, 41] may offer an opportunity to encourage PID patient screening in the existing global acute flaccid paralysis surveillance system by including stool sample results from all newly identified PID patients in global poliovirus surveillance reporting. As long as OPV use continues, a comprehensive screening system would require continued periodic screening of PID patients for poliovirus and regular follow-up of excretors to track persistent infections. After successful OPV cessation, no live poliovirus circulation should occur, such that screening for poliovirus upon diagnosis of every new PID patient should suffice to identify any iVDPV infections. However, the reality of PID under-diagnosis and the diagnostic delay [42–44] represent important obstacles we must address to achieve high sensitivity of iVDPV screening. Given our findings of risks of problematic outbreaks associated with prolonged iVDPV excretors who do not stop excretion within two years of OPV cessation in countries with high poliovirus transmissibility, we emphasize the urgency of efforts to develop effective PAVDs and expand screening efforts for asymptomatic long-term iVDPV excretors.

## Supplementary material

For supplementary material accompanying this paper visit http://dx.doi.org/10.1017/S0950268816002302. click here to view supplementary material
