# Supplementary material for: Comprehensive screening for immunodeficiency-associated vaccine-derived poliovirus: an essential oral poliovirus vaccine cessation risk management strategy
Source: Epidemiol Infect. 2016 Oct 20;145(2):217–26. doi: 10.1017/S0950268816002302 (PMC5197684; doi:10.1017/S0950268816002302)
Supplement: Supplementary file 1 [file S0950268816002302sup001.docx]

**Epidemiology and Infection**

**Comprehensive screening for immunodeficiency-associated vaccine-derived poliovirus: an essential OPV cessation risk management strategy**

**R.J. Duintjer Tebbens and K.M. Thompson**

**SUPPLEMENTARY MATERIAL**

This file provides technical details and additional results. We refer to the main paper for abbreviations not separately defined in this file.

***S1. DES model details and uncertainty analysis***

The DES model of global long-term iVDPV prevalence differentiates between common variable immunodeficiency disease (CVID) and all other PIDs that may lead to reduced ability to clear OPV infections (oPIDs)[[1](#_ENREF_1)], which represent a wide spectrum of B-cell related conditions for which insufficient data exist for further differentiation. The prevalence of long-term iVDPV excretors depends on the overall incidence of CVID and oPIDs per birth, the average age of PID onset, the exposure to OPV viruses, the proportion of CVID and oPID patients pre-disposed to develop a long-term infection (if infected with OPV after PID onset and surviving long enough), and the survival rates of CVID and oPID patients in different countries (which depend on intravenous immunoglobulin treatment rates and hygienic conditions). Any PID patient excreting an iVDPV after cessation of OPV of the excreted serotype results in potential iVDPV introductions into the general population based on assumed contact rates with the general population (i.e., beyond presumed immune close contacts), which we determine randomly for each excretor.[[2](#_ENREF_2)]

To consider the role of major uncertainties in the DES model,[[1](#_ENREF_1)] we performed 1,000 iterations of the DES model, with key uncertain model inputs randomly and independently drawn from the uncertainty distributions in Table S1. For model inputs with values that vary by characteristic (e.g., type of PID, R_0_ value), we fully correlate these values by drawing a single random uniform number to determine realizations for each characteristic based on the inverse cumulative distribution functions.[[3](#_ENREF_3)] For inputs with a lognormal distribution with lower limit x, median y, and 99.5^th^ percentile z, we compute the parameters μ and σ of the lognormal distribution as:

μ = Ln(y-x)

σ = min_σ_|F^-1^_μ,σ_(0.995) – (z-x)|

In the latter equation, F^-1^_μ,σ_ represents the inverse cumulative lognormal distribution with parameters μ and σ, and we find the σ that minimizes the right hand side of the equation using the goal seek function of MS Excel^TM^. To sample from a lognormal distribution with parameters μ and σ in the DES model that we implemented in JAVA^TM^, we first approximate the inverse cumulative normal distribution function (probit function) φ_μ,σ_ using the explicit approximation proposed by Soranzo and Epure.[[4](#_ENREF_4)] For a given random standard uniform realization *u*, the corresponding lognormal realization *l* then equals:

*l* = x + exp(μ+ σ×φ_μ,σ_ (u))

Based on the 1,000 DES model iterations with input uncertainty, we report the distributions of the time until the last iVDPV excretor stops excreting in each income level. We compare these distributions with the distributions of the set of 1,000 DES model iterations used for the global model base case,[[3](#_ENREF_3)] which keep all DES model at the base case values from Table S1. We also rank how much each uncertain DES model input contributes to the overall uncertainty in the time until the last excretor stops excreting based on the rank (Spearman) correlation.[[3](#_ENREF_3)] Rank correlation values closer to 1 indicate a stronger increasing relationship between the model input and the time until the last iVDPV excretors stops, while values closer to -1 indicate a stronger decreasing relationship, and values near 0 indicate little influence of the model input on the time until the last iVDPV excretor stops.[[3](#_ENREF_3), [5](#_ENREF_5), [6](#_ENREF_6)]

Figure S1 shows the distributions of the time when the last long-term iVDPV excretor stops excreting (through spontaneous recovery from the infection or death) with or without accounting for uncertainty in the model inputs from Table S1. Due to truncation at the end of the analytical time horizon in 2053, all excretors that continue through 2053 show up in the last data point, accounting for the apparent spike in the distributions for the year 2053. The distributions without DES model input uncertainty (Figure S1a) reflect only stochastic variability associated with the large number of random events inherent in the DES model. Although the occurrence of random events in the DES model accounts for much uncertainty in future iVDPV risks, we find substantially wider distributions if we include DES model input uncertainty (Figure S1b). With input uncertainty, the probability of very long times until the last iVDPV excretors stops excreting increases in each income level and thus also globally. Without input uncertainty, the mean time when the last iVDPV excretor in the world stops excreting equals early 2028 (standard deviation approximately 6.0 years), and with input uncertainty the mean time extends to mid-2032 (standard deviation approximately 9.8 years).

***S2. Global model details and characteristics of OPV restart iterations***

The global model results based on 100 stochastic iterations suggested very small benefits of the PAVD40% scenario, but an increase in incremental net benefits for the PAVD90% scenario of approximately $0.5 billion compared to the base case incremental net benefits of $16-17 billion.[[2](#_ENREF_2)] We subsequently performed 1,000 stochastic iterations of the global model to obtain more robust estimates of the OPV restart probability and incremental net benefits for the current plan, which resulted in 57 iterations with an OPV restart (OPV restart fraction 0.057, 95% confidence interval 0.043 to 0.071).[[3](#_ENREF_3)] Compared with the 2 OPV restart iterations in the original set of 100 stochastic iterations, these 57 iterations provide a larger and more representative sample of conditions that may lead to an OPV restart, including those related to iVDPVs. To optimally represent the space of possible outcomes while limiting the computational demands for this study, we selected a set of 120 iterations that comprises all 57 OPV restart iterations and 63 iterations without OPV restart (we used the first 63 iterations, the choice of which did not significantly affect the results).[[3](#_ENREF_3)] We then compute expected outcomes by giving all 57 OPV restart iterations a weight of 1/1000 (i.e., together they represent 57 out of 1,000 possible iterations) and all iterations without an OPV restart a weight of (1/63)×943/1000 (i.e., together they represent the remaining (1000-57) out of 1,000 possible iterations).

Table S2 summarizes selected characteristic of all 57 global model iterations (out of 1,000 total iterations performed) that resulted in an OPV restart with the baseline IPV5 policy and no PAVDs. Although most triggering events that ultimately resulted in an OPV restart occurred during the first 10 years after OPV cessation, due to the assumed aggressive outbreak response with mOPV (during the first 5 years after OPV cessation of each serotype) and IPV (thereafter), it typically took many years until the cumulative number of polio cases (since 2016) exceeded 50,000 and resulted in the OPV restart. Consistent with the ordering of R_0_ values and PIRs for WPVs and VDPVs by serotype (1>2>3),[[7](#_ENREF_7)] 67%, 24%, and 9% of OPV restarts related to uncontrolled outbreaks of serotype 1, 2, and 3, respectively. In part due to the assumption of sufficient OPV intensification before OPV cessation of each serotype to prevent all cVDPV outbreaks,[[2](#_ENREF_2), [8](#_ENREF_8)] OPV restarts overwhelmingly related to poliovirus reintroductions from long-term iVDPV excretors (91% of OPV restarts), including 7 OPV restarts (12%) involving a reintroduction from a long-term iVDPV excretor infected during an mOPV outbreak response SIA after OPV cessation. Long-range exportations of outbreak virus or viruses related to mOPV used for outbreak response played an essential role in perpetuating outbreaks in only 5 OPV restarts (9%), because typically the triggering event already led to uncontrolled outbreaks in the block experiencing the event. We observed two distinct but common types of scenarios that together accounted for 90% of all OPV restarts. The first type accounted for 26 OPV restarts (46%) and involved prolonged and chronic iVDPV excretors that introduced the virus at least two years after homotypic OPV cessation into populations with an R_0_ between 6-9 for WPV1 (which serves as our measure of inherent poliovirus transmissibility of all poliovirus serotypes and reversion stages, since they directly relate to the R_0_ for WPV1 [[7](#_ENREF_7)]), including 16 (28%) between 5-12 years after homotypic OPV cessation. In this type of OPV restart, the late occurrence of the event after OPV cessation and associated decrease in population immunity to transmission led to a failure to control the outbreaks within the 5-year window of relatively safe mOPV use for outbreak response. The second type accounted for 25 OPV restarts (44%) and involved prolonged iVDPV excretors that introduced the virus within the first five years after homotypic OPV cessation in populations with an R_0_ for WPV1 between 10-13, such that even aggressive outbreak response could not re-interrupt transmission. This type included 20 (35%) iVDPV introductions within the first two years in populations with an R_0_ for WPV1 of 13. As shown in Table S2, high RI coverage with IPV-only in the populations of the triggering event did not prevent OPV restarts because of the ability of fecal-oral transmission to continue despite immunity induced by IPV alone.

**Table S1: Assumed uncertainty distributions of key inputs in the DES model[**[**1**](#_ENREF_1)**]**

| **DES model input** | **Base case estimate** | **Assumed uncertainty distribution^a^** |
| --- | --- | --- |
| PID predisposition probability per birth   - CVID - oPID | 1/32,000  1/8,500 | Trian(1/100,000;1/32,000;1/10,000)  Trian(1/20,000;1/8,500;1/2,000) |
| Potential^b^ long-term excretion probability   - Prolonged, CVID - Prolonged, oPID - Chronic, CVID - Chronic, oPID | 0.01  0.01  0.005  0 | Lognorm(0.01;0.001;0.2)  Lognorm(0.01;0.001;0.2)  Lognorm(0.005;0.001;0.1)  Lognorm(0.001;0;0.005) |
| Relative monthly death rate vs. baseline   - Untreated - R_0_ of WPV1 is 4 or 5 - R_0_ of WPV1 is 6 - R_0_ of WPV1 is 7 - R_0_ of WPV1 is 8 - R_0_ of WPV1 is 9 - R_0_ of WPV1 is 10 - R_0_ of WPV1 is 11 - R_0_ of WPV1 is 12 - R_0_ of WPV1 is 13 | 5  1  5  10  20  25  35  40  45  50 | Unif(2.5;7.5)^c^  Unif(0.5;1.5)  Unif(2.5;7.5)  Unif(5;15)  Unif(10;30)  Unif(12.5;37.5)  Unif(17.5;52.5)  Unif(20;60)  Unif(22.5;67.5)  Unif(25;75) |
| Monthly PID onset probability^d^   - CVID - oPID | 1/300  1/24 | Trian(1/360;1/300;1/120)  Trian(1/120;1/24;1/3) |
| Increase in all OPV exposure rates^e^ | 1 | Unif(0.5;1.5) |
| Average duration of iVDPV infection (years)^f^   - Prolonged - Chronic | 2  15 | Unif(1;3)  Unif(7;15;23) |
| Relative probability of long-term OPV infection if treated vs. not treated | 0.5 | Unif(0;1) |

**Abbreviations:** CVID, common variable immunodeficiency disease; DES, discrete-event simulation; iVDPV, immunodeficiency-associated vaccine-derived poliovirus; oPID, other PID; OPV, oral poliovirus vaccine; PID, primary immunodeficiency disease

**Notes:**

^a^ Trian(x,y,z) indicates a triangular uncertainty distribution with lower limit x, mode y, and upper limit z; Lognorm(x,y,z) indicates a lognormal uncertainty distribution with lower limit x, median y, and 99.5^th^ percentile z; Unif(x,y) indicates a uniform uncertainty distribution with lower limit x and upper limit y

^b^”Potential” refers to the ability to develop a long-term infection, conditional on an OPV infection after PID onset and sufficiently long survival after the infection to become of prolonged or chronic excretor

^c^ All uncertainty distributions for this model input assumed to range from -50% to +50% of the base case estimate

^d^ Model input corresponds to 1 divided by 12 times the average age of onset in years, i.e., the base case values imply average ages at onset of 25 (CVID) and 2 (oPID) years

^e^ Multiplier applies to primary, secondary OPV exposure rates with or without diagnosis in all income levels and for all immunization schedules

^f^ Applied using monthly probabilities as described,[30] which cuts off prolonged infections at 5 years to effectively slightly reduce average duration

**Table S2: Characteristics of 57 global model iterations that resulted in an OPV restart with the IPV5 policy and no PAVDs**

| **Characteristic** | **Number of iterations (% of OPV restarts)** | **Probability, based on 1,000 iterations** |
| --- | --- | --- |
| All OPV restarts | 57 (100) | 0.0057 |
| Year of OPV restart   - 2030-2034 - 2035-2039 - 2040-2044 - 2045-2049 - 2050 | 14 (25)  14 (25)  19 (33)  8 (14)  2 (4) | 0.014  0.014  0.019  0.008  0.002 |
| Year of primary triggering event^a^   - 2018-2019 - 2020-2024 - 2025-2029 - 2030-2039 - 2040-2049 | 21 (37)  26 (46)  5 (9)  2 (4)  3 (5) | 0.021  0.026  0.005  0.002  0.003 |
| Serotype of primary triggering event   - Serotype 1 - Serotype 2 - Serotype 3 | 38 (67)  14 (25)  5 (9) | 0.038  0.014  0.009 |
| Nature of primary triggering event   - iVDPV - Unintentional release from laboratory or intentional release | 52 (91)  5 (9) | 0.052  0.005 |
| Secondary triggering event^b^   - None (i.e., transmission continued regardless) - iVDPV excretor infected by mOPV used for outbreak response - Long-range^c^ outbreak virus exportation - Longe-range^c^ exportation of virus related to mOPV used for outbreak response | 45 (79)  7 (12)  4 (7)  1 (2) | 0.045  0.007  0.004  0.001 |
| R_0_ for WPV1 in population of primary triggering event   - 6 or 7 - 8 or 9 - 10 or 11 - 12 or 13 | 13 (21)  16 (28)  6 (11)  22 (39) | 0.013  0.016  0.006  0.022 |
| POL3 coverage (%) in population of primary triggering event   - 30 - 60 - 90 - 98 | 5 (9)  26 (46)  19 (33)  7 (12) | 0.005  0.026  0.019  0.007 |

**Abbreviations:** iVDPV, immunodeficiency-associated vaccine-derived poliovirus; mOPV, monovalent OPV; OPV, oral poliovirus vaccine; PAVD, polio antiviral drug; POL3, coverage with 3 or more non-birth polio routine immunization doses; R_0_, basic reproduction number; WPV1, serotype 1 wild poliovirus

^a^ We define a primary triggering event defined as a poliovirus reintroduction that triggers an outbreak and/or new events (exportations of outbreak virus, exportations of virus related to mOPV used for outbreak response, or newly infected iVDPV excretor by mOPV used for outbreak response) that result ultimately in over 50,000 polio cases since 2016

^b^ We define a secondary triggering event as an extra-block outbreak-related or mOPV-related virus exportation or poliovirus reintroduction from a newly infected iVDPV excretor by mOPV used for outbreak response, without which transmission would have stopped globally

^c^ Defined as an effective importation into a subpopulation of a different blocks than the exporting subpopulation

**Figure S1: Probability distribution functions of the time until the last immunodeficiency-associated vaccine-derived poliovirus (iVDPV) excretor stops excreting by income level, with or without the discrete-event simulation (DES) model input uncertainty specified in Table A1.**

**(a) DES model inputs at their base case values**

**
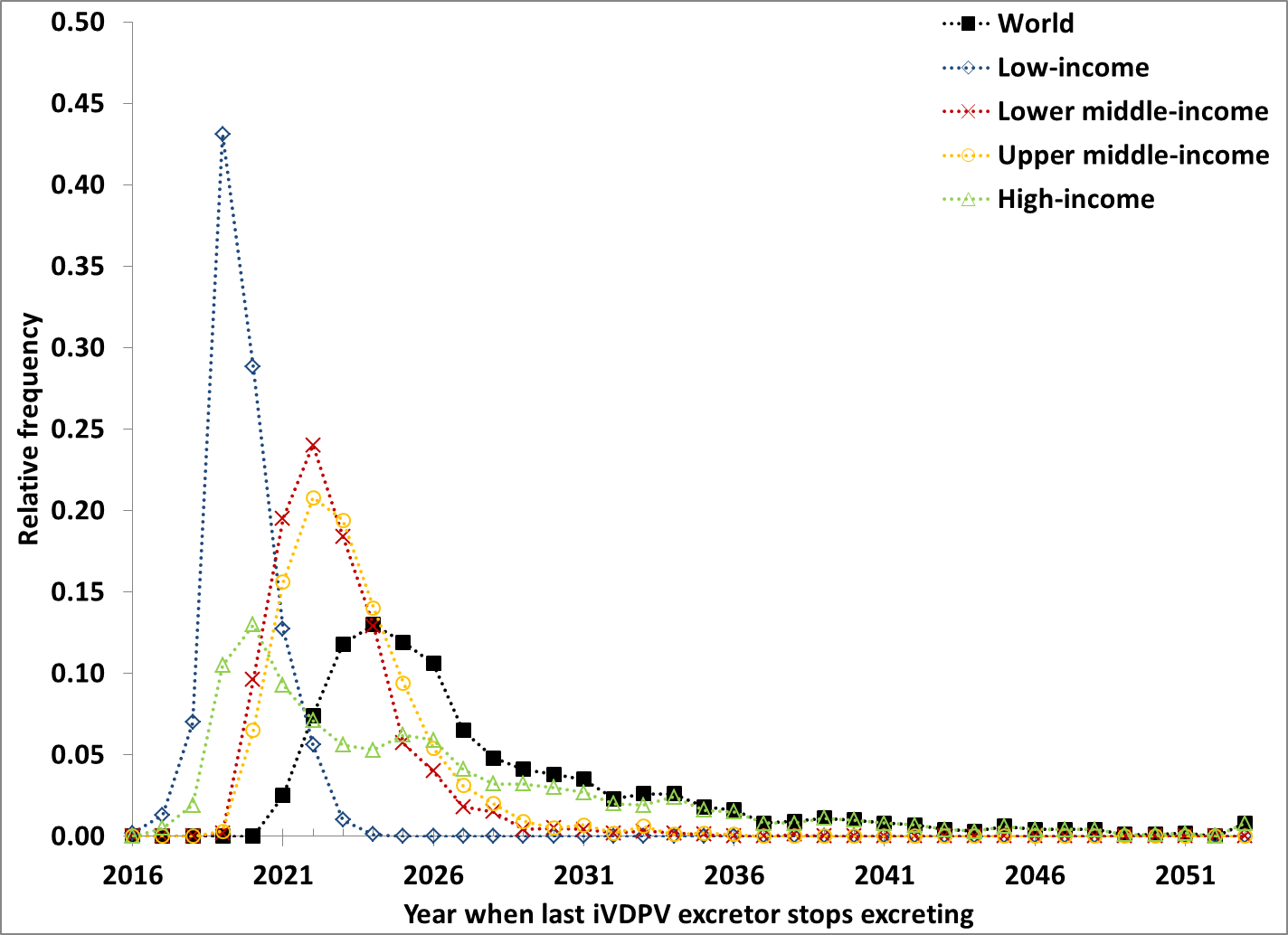
**

**(b) DES model input uncertainty included**

**
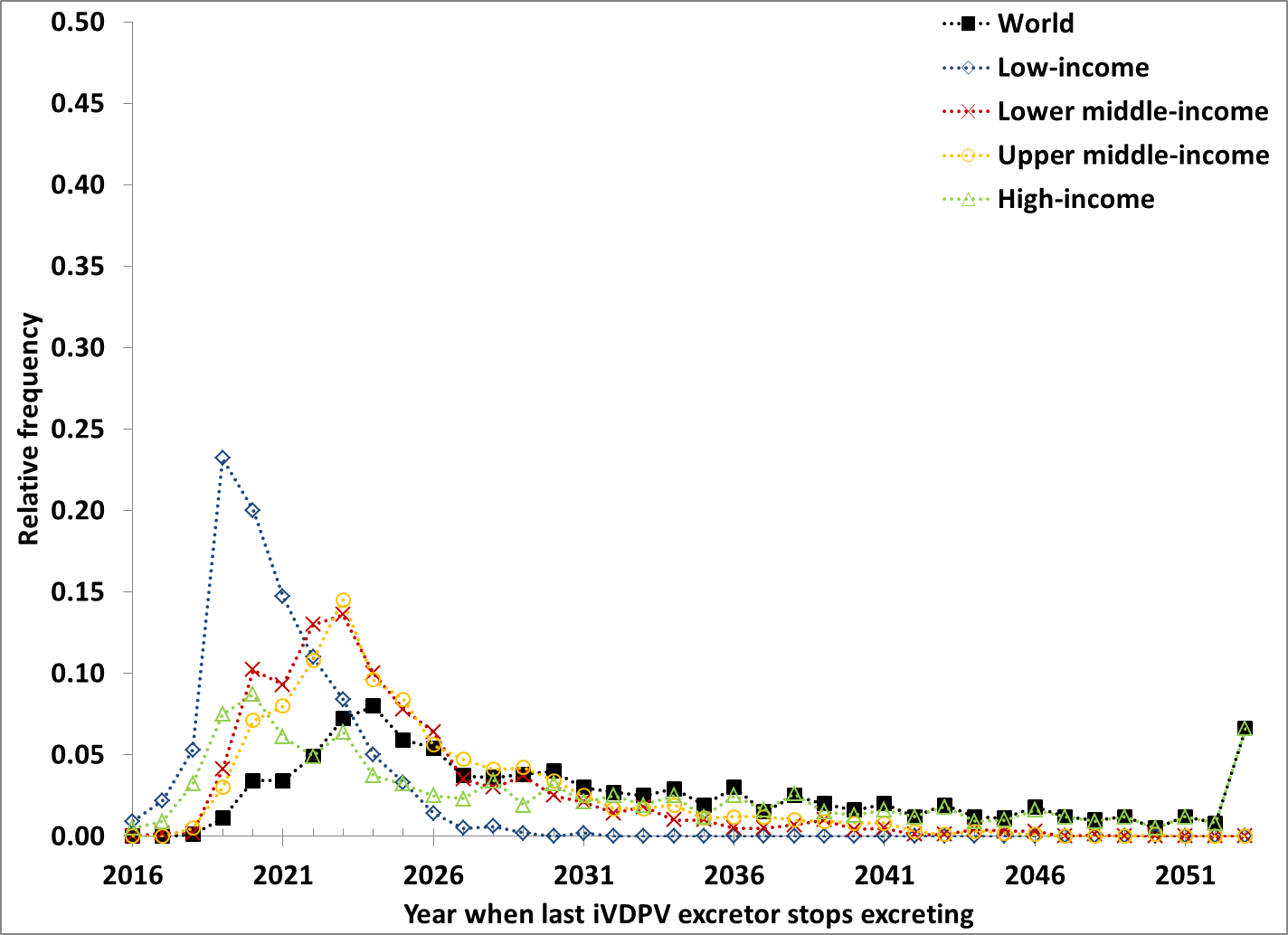
**

**REFERENCES**

1. **Duintjer Tebbens RJ, Pallansch MA, Thompson KM.** Modeling the prevalence of immunodeficiency-associated long-term vaccine-derived poliovirus excretors and the potential benefits of antiviral drugs. *BMC Infectious Diseases* 2015; **15**: 379.

2. **Duintjer Tebbens RJ, et al.** An economic analysis of poliovirus risk management policy options for 2013-2052. *BMC Infectious Diseases* 2015; **15**: 389.

3. **Duintjer Tebbens RJ, Thompson KM.** Uncertainty and sensitivity analysis of cost assumptions for global long-term poliovirus risk management (in press). *Journal of Vaccines and Vaccination* 2016.

4. **Soranzo A, Epure E.** Very simply explicitly invertible approximations of normal cumulative and normal quantile function. *Applied Mathematical Sciences* 2014; **8**: 4323-4341.

5. **Duintjer Tebbens RJ, et al.** Uncertainty and sensitivity analyses of a dynamic economic evaluation model for vaccination programs. *Medical Decision Making* 2008; **28**: 182-200.

6. **Duintjer Tebbens RJ, et al.** Uncertainty and sensitivity analyses of a decision analytic model for post-eradication polio risk management. *Risk Analysis* 2008; **28**: 855-876.

7. **Duintjer Tebbens RJ, et al.** Characterizing poliovirus transmission and evolution: Insights from modeling experiences with wild and vaccine-related polioviruses. *Risk Analysis* 2013; **23**: 703-749.

8. **Duintjer Tebbens RJ, Thompson KM.** Managing the risk of circulating vaccine-derived poliovirus during the endgame: Oral poliovirus vaccine needs. *BMC Infectious Diseases* 2015; **15**: 390.
